# Three Diverse Granule Preparation Methods for Proteomic Analysis of Mature Rice (*Oryza sativa* L.) Starch Grain

**DOI:** 10.3390/molecules27103307

**Published:** 2022-05-21

**Authors:** Zachary Provost, Ella Olivia Hansen, Morgan Viola Lynds, Barry S. Flinn, Zoran Minic, Maxim V. Berezovski, Illimar Altosaar

**Affiliations:** 1 Department of Biochemistry, Microbiology and Immunology, University of Ottawa, Ottawa, ON K1H 8M5, Canada; zacharyprovost@icloud.com (Z.P.); liv.live47@gmail.com (E.O.H.); mlynd072@uottawa.ca (M.V.L.); bsflinn@gmail.com (B.S.F.); 2 Department of Chemistry and Biomolecular Sciences, University of Ottawa, Ottawa, ON K1N 6N5, Canada; zminic@uottawa.ca (Z.M.); maxim.berezovski@uottawa.ca (M.V.B.); 3 John L. Holmes Mass Spectrometry Facility, Faculty of Science, University of Ottawa, Ottawa, ON K1N 6N5, Canada; 4 Proteins Easy Corp, 75 Campus Drive, Kemptville, ON K0G 1J0, Canada

**Keywords:** rice, starch grain (SG), amyloplast, starch granule, plastoskeleton, proteome, starch granule-associated protein (SGAP), septum-like structure (SLS)

## Abstract

Starch is the primary form of reserve carbohydrate storage in plants. Rice (*Oryza sativa* L.) is a monocot whose reserve starch is organized into compounded structures within the amyloplast, rather than a simple starch grain (SG). The mechanism governing the assembly of the compound SG from polyhedral granules in apposition, however, remains unknown. To further characterize the proteome associated with these compounded structures, three distinct methods of starch granule preparation (dispersion, microsieve, and flotation) were performed. Phase separation of peptides (aqueous trypsin-shaving and isopropanol solubilization of residual peptides) isolated starch granule-associated proteins (SGAPs) from the distal proteome of the amyloplast and the proximal ‘amylome’ (the amyloplastic proteome), respectively. The term ‘distal proteome’ refers to SGAPs loosely tethered to the amyloplast, ones that can be rapidly proteolyzed, while proximal SGAPs are those found closer to the remnant amyloplast membrane fragments, perhaps embedded therein—ones that need isopropanol solvent to be removed from the mature organelle surface. These two rice starch-associated peptide samples were analyzed using nano-liquid chromatography–tandem mass spectrometry (Nano-HPLC-MS/MS). Known and novel proteins, as well as septum-like structure (SLS) proteins, in the mature rice SG were found. Data mining and gene ontology software were used to categorize these putative plastoskeletal components as a variety of structural elements, including actins, tubulins, tubulin-like proteins, and cementitious elements such as reticulata related-like (RER) proteins, tegument proteins, and lectins. Delineating the plastoskeletal proteome begins by understanding how each starch granule isolation procedure affects observed cytoplasmic and plastid proteins. The three methods described herein show how the technique used to isolate SGs differentially impacts the subsequent proteomic analysis and results obtained. It can thus be concluded that future investigations must make judicious decisions regarding the methodology used in extracting proteomic information from the compound starch granules being assessed, since different methods are shown to yield contrasting results herein. Data are available via ProteomeXchange with identifier PXD032314.

## 1. Introduction

In plants, energy is stored in the form of starch, an accumulation of the glucose polymers amylose and amylopectin. Starch can either be transitory—meaning that it is synthesized in the aerial tissue during the day and is broken down during the night to sustain cellular metabolism [1]—or storage, where starch is sequestered into non-photosynthetic organs for long-term use in subcellular structures called amyloplasts. Storage starch grains (SGs) can be either simple or compound, whereby they are either composed of a single discrete unit or multiple subunits. Many monocot crop species produce simple starch granules—maize (*Zea mays*), sorghum (*Sorghum bicolor*), barley (*Hordeum vulgare*), and wheat (*Triticum aestivum*) are all examples of this mode of carbon sequestration [2]. In rice and oat, multifaceted subunits called starch granules are packaged into a higher order of structure and agglomerate or coalesce into compound SGs [3]. This study made use of rice as a model for optimizing the SG preparation method and includes an analysis and comparison of the three techniques used.

The rice amyloplast is proposed to be composed of several distinct components: an outer envelope membrane (OEM) and an inner envelope membrane (IEM), which enclose an intermembrane space (IMS); and a septum-like structure (SLS) that forms between apposing granule surfaces. The prevailing wisdom regarding compound granule agglomeration hinges on the role of the IEM. The IEM is hypothesized to form a mold in which starch molecules are deposited, forming the characteristic polyhedral granule [4,5], although the molecular mechanism by which the IEM is proposed to form these molds has not yet been described. The hypothesis of an underlying protein scaffolding is inferred from proteins that are involved in fission and septum development in the amyloplast [4,6]. The Brittle1 protein (BT1) is an ADP-glucose transporter that localizes to the SLS within the rice SG and may be responsible for septum development in the rice amyloplast [7,8,9] and starch granule channels in maize [10]. Plastid division proteins and other SLS proteins (BT1) are present in the maize endosperm and may play a role in fusing the IEM to form the SLS between starch granules during endosperm development [6,11,12]. However, less is known about the underlying plastoskeletal structure holding together this quaternary glucan deposit in the mature rice SG.

The missing pieces of information regarding the SG scaffolding or plastoskeleton may be present in the starch granule-associated protein (SGAP) proteome [13,14]. As such, the preparation of SGs and the analysis of the SGAP [15,16] of the rice starch granule is the focus of this study. Mature rice kernels were used in an effort to eliminate any bias that may be introduced by the commercial processing of rice starch [15]. In this study, three methods of starch granule preparation from mature rice kernels (dispersion, microsieve, and flotation) were used, following which phase separation using two different approaches (trypsin shaving and isopropanol solubilization of residual peptides) [17,18] was performed. Starting with granules prepared as much as practically possible, it was hypothesized that trypsinization would yield a proteome loosely associated with the granule surface (distal) and the subsequent alcohol ‘scrubbing’ of the trypsinized surface would identify peptide domains more tightly associated with the granule surface (proximal), respectively. In other words, this new term ‘distal proteome’ can be used to refer to SGAPs loosely tethered to the amyloplast, ones that can be rapidly proteolyzed, while proximal SGAPs are those found closer to the remnant amyloplast membrane fragments, and perhaps even embedded therein, SGAP proteins that need isopropanol solvent to be removed from the mature lipid-containing organelle surface. A mass spectrometry-based survey of the six types of peptide samples permitted the partial characterization of rice starch granules.

## 2. Results

**Figure 1:**
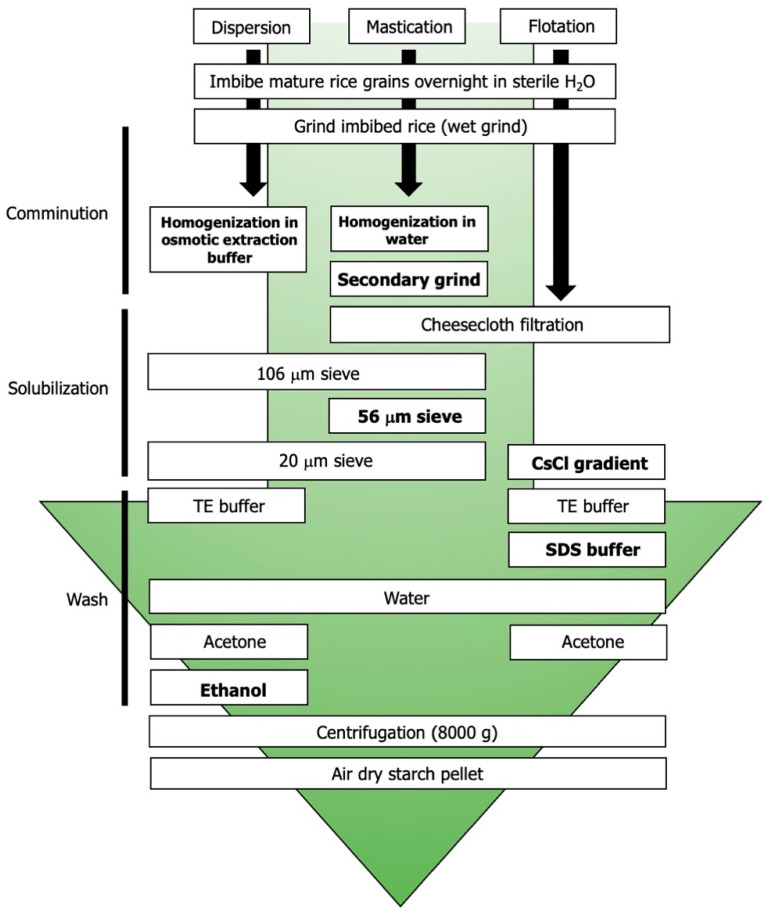
The various preparation methods used to compare starch granules in this study. Biochemical isolation steps that were unique to the preparation method are represented in boldface font.

**Figure 2:**
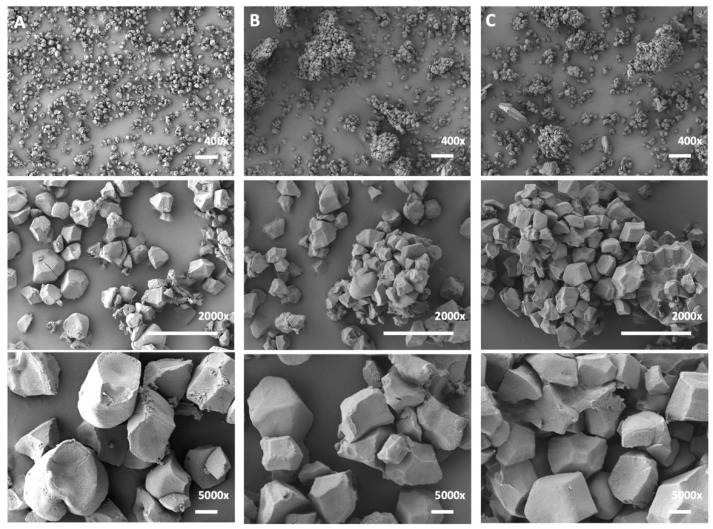
Images of rice starch granules prepared by scanning electron microscope (SEM) images. (**A**) Dispersion-prepared granules. (**B**) Microsieve-prepared starch granules. (**C**) Flotation-prepared starch granules. 400× magnification, bar = 10 μm; 2000× magnification, bar = 10 μm; 5000× magnification, bar = 1 μm.

For this study, three rice preparation methods were employed (Figure 1) and each sample was sequentially trypsinized and treated with isopropanol with the aim of comparing their impact on the accessibility and extractability of SGAPs. All three preparation methods used efficiently disrupted the starch grain. Scanning electron microscope (SEM) imaging confirmed that all three starch preparation methods produced intact polyhedral starch granules (Figure 2). Dispersion preparation produced the greatest amount of separation among the granules (Figure 2A). Microsieve preparation produced individual granules but some agglomeration of granules remained visible (Figure 2B). Using flotation to prepare starch granules was the least effective method, as there were both agglomerated starch granules and intact SGs (Figure 2C) present in the samples. Granules prepared by all three methods were free of remaining protein bodies. The dispersion method produced granules with less fragmentation than the other two methods (Figure 2A) but the granules did not have the same faceted edges as the granules prepared via flotation (Figure 2C).

A proteomic analysis of the starch granule samples was performed. The numbers of total and uncharacterized peptides identified in each of the six starch samples by mass spectrometric analysis are shown in Table 1. Since the general content of the rice SGAP has been established [16], known proteins (excluding structural proteins) were subtracted from the analyses. The abundant proteins present in the samples were primarily glutelins and other starch metabolism proteins (Table 2) and were significantly abundant in all six samples. For the purpose of this study, these protein groups were eliminated from further analysis. The total mass spectra datasets for each preparation technique are available in the Supplementary Information (Datasets S1–S6).

Uncharacterized peptides were present in all three samples (Supplementary Materials, Tables S1–S6). No protein data were returned for these peptides following SEQUEST analysis against the UniProt database, and they are annotated only by locus ID. All uncharacterized peptides can be found in Datasets S1–S6, which include the UniProt accession ID, protein description, sum PEP score (posterior error probability), percent protein coverage, and number of peptide hits. Uncharacterized peptides in the mass spectrometric datasets were analyzed using the NCBI Web CD-Search Tool (www.ncbi.nlm.nih.gov/Structure/bwrpsb/bwrpsb.cgi accessed on 4 October 2019) of the NCBI Batch Conserved domain database [19] and PantherDB v.14.1 [20] to obtain data on uncharacterized peptides (Supplementary Materials, Tables S1–S6). Most uncharacterized peptides have no assigned function. To obtain a broad view of what types of proteins are present in the uncharacterized sets of each of the six samples, Gene Ontology (GO) Enrichment Analysis powered by Panther (geneontology.org accessed on 4 October 2019) [20] was performed. GO analysis assigns roles to proteins within three main categories: molecular function, cellular component, and biological process. To calculate the number and percentages of proteins belonging to each GO category and to obtain a comprehensive interpretation of the common protein functions and their GO functions, the accession IDs of the uncharacterized peptides (Supplementary Materials, Tables S1–S6) were submitted for GO analysis using PantherDB v.14.1. Most of the peptides in the distal proteome (396 proteins) and proximal amylome (82 proteins) of the dispersion-method-prepared starch granules had no known role in any biological process or a known molecular function (Supplementary Materials, Figure S3).

It was found that the use of diverse starch granule extraction methods can identify the core proteome. A common distal proteome was compiled using the mass spectrometric data obtained from all aqueous samples from all three starch preparation methods. The distinction between the distal and proximal proteomes is based on the rationale that proteins which are easily removeable from the starch granule by trypsin digest would also be more distal to the starch granule [17]. Similarly, it was hypothesized that the core amylome IDs were isolated by treating the trypsinized starch granule samples with isopropanol to remove the tightly bound proteins—the rationale being that these more tightly bound proteins would be more closely associated with the starch granule (membrane?) than the loosely bound proteins more accessible to trypsin digestion. Venn diagram analysis software InteractiVenn (interactivenn.net) [21] assembled common proteomes among all relevant permutations of the mass spectrometry datasets (Supplementary Materials, Figure S1). A common set of 11 unique proteins was found among the aqueous samples collected and analyzed from all three starch preparation methods (Table 2; Supplementary Materials, Figure S1A). Similarly, a common set of 31 unique proteins was found among the isopropanol-solubilized samples collected and analyzed from the three starch granule preparation methods (Table 2; Supplementary Materials, Figure S1B). There were three proteins common to the distal proteome of the amyloplast and amyloplastic proteome: sorbitol dehydrogenase, pyruvate dikinase, and glutelin type-2A (Table 2; Supplementary Materials, Figure S1C). Of the remaining nine proteins detected in the enzyme digests, three were glutelins, one prolamin, one globulin, three starch biosynthesis proteins, and one stress-response protein. SLS-localizing protein Brittle1 (BT1) [22] was present in the distal proteomes obtained from all three starch preparation methods (Table 2).

Twenty-eight remaining proteins made up the amylome. Of these, five were glutelin isoforms, three were transcriptional/translational machinery proteins, two were seed storage proteins, and one was a membrane structural protein. The remaining 17 were related to starch biosynthesis and sucrose metabolism. They included sucrose synthase, granule-bound starch synthase, branching enzyme, ADP-glucose pyrophosphorylase, pullulanase, α-glucosidase, and α-1,4-glucan phosphorylase. Other carbohydrate-metabolism-related proteins identified included sorbitol dehydrogenase, glyceraldehyde phosphate, fructose-bisphosphate aldolase, and orthophosphate dikinase. BT1 was not found in the core proximal amylome.

Both known and novel structural proteins and a novel carbohydrate-binding protein were identified in the rice SG samples (Table 3). To correlate proteomic trends with starch and protein extraction methods, Venn diagrams were compiled (Supplementary Materials, Figure S2) using both Proteome Discoverer output tables for each of the starch granule preparation methods (Datasets S1–S6). This analysis parsed proteins unique to each dataset. The results were as follows: dispersion-distal (1171 unique proteins), dispersion-proximal (164), microsieve-distal (18), microsieve-proximal (13), flotation-distal (8), and flotation-proximal (3). The identities of the unique proteins are available (Datasets S7–S12).

A large amount of data was obtained following trypsin shaving and isopropanol treatment of the dispersion-prepared granules (Table 1) and so analysis was restricted to the uncharacterized protein dataset. The distal proteome obtained was smaller than the proximal amylome, and for both datasets, the major groups included were seed storage (glutelins), starch biosynthesis (starch synthase), and metabolism (fructose-bisphosphate aldolase 3, glyceraldehyde-3-phosphate dehydrogenase). There were no identifiable structural proteins in the top 50 uncharacterized hits for either the distal or proximal amylomes (Supplementary Materials, Tables S1 and S2). However, in the distal proteome, there was one chloroplast inner envelope protein (Q7XD45). Most protein hits were associated with starch biosynthesis, metabolism, and seed storage proteins. There were 38 proteins shared between the two proteomes. Similar results were observed for the lists obtained from the flotation-prepared starch granules, although no proteins were shared between the trypsin-shaved and isopropanol-solubilized samples (Table 1; Supplementary Materials, Tables S5 and S6).

Each starch sample contained uncharacterized peptides, but database screening assigned functions to most of these peptides (Supplementary Materials, Tables S7–S12). The data presented in these tables were primarily the result of batch analysis in PantherDB. Peptides that could not be identified using this method were analyzed with the NCBI Batch Web CD-Search Tool to find conserved domains. Some accession IDs from the mass spectrometric data did not match up with a recognized protein and so were designated unknown.

Actins and tubulins were present in the proteome of dispersion-prepared starch granules. Four actin proteins and ten tubulin proteins (three alpha chains and four beta chains) were detected in the distal proteome of dispersion-prepared starch granules. There were two actin-depolymerizing factors (ADFs) in the same proteome: ADF-2 (Q9AY76) and ADF-3 (Q84TB6) (Table 3). Plant ADFs are proteins with low molecular weights (16–20 kilodaltons) which promote actin cytoskeleton turnover rates by acting together with profilin to sever actin filaments [23]. There were four actin proteins and four tubulin proteins (three alpha chains and one beta chain) in the proximal amylome of dispersion-extracted starch granules. One tubulin (Q10PW2) was found in both the distal and proximal amylome. There were three actin-depolymerizing factors in the same proteome: ADF-3 (Q84TB6), ADF-4 (Q84TB3), and ADF-7 (Q0DLA3) (Table 3). No structural proteins were identified in the aqueous supernatant of trypsin-shaved microsieve-prepared starch granules, nor in the trypsin-shaved or isopropanol-solubilized fractions from flotation-extracted starch granules. One actin and three tubulins were found in the isopropanol-solubilized fraction of microsieve-prepared starch granules (Table 4). Uncharacterized proteins were found in the distal and proximal extracts of dispersion- and microsieve-prepared starch granules.

Peptides which were uncharacterized were either run through Retrieve/ID mapping via UniProt to obtain putative function [24], or through the NCBI Batch Conserved domain database [19,25] within the Web CD-Search Tool. The latter was preferred as it contains the most recently updated proteome database (March 26th, 2020) and was used to identify structural domains in the unknown proteins. Peptides from putative carbohydrate-binding proteins were analyzed using the Carbohydrate Active enZYmes (CAZy) domain database to confirm the presence of carbohydrate-binding module (CBM) domains [26] or using the PantherDB v.14.1 Classification System (PantherDB) to identify the functional domains of the uncharacterized proteins (Table 3 and Table 4).

Novel uncharacterized peptides were found in the aqueous fraction of dispersion-prepared starch granules treated with trypsin. Most uncharacterized peptides were found in dispersion-prepared granules. Some of these peptides could not be identified using UniProt Retrieve/ID mapping analysis but were available in the NCBI database due to the update of the rice proteome (26 March 2020). The bulk of newly identified proteins were found in dispersion-prepared granules. Some of these proteins were present in the UniProt database but were available in the NCBI database due to the update of the rice proteome (26 March 2020). Proteins that had not yet been annotated by NCBI were examined based on the presence of a conserved domain. Domain conservation is qualified by multiple sequence alignments of related proteins across multiple species that reveal the same or similar amino acid patterns [19]. Peptides from putative starch-binding proteins were analyzed using the CAZy database. A diverse list of proteins that were categorized by domain analysis was compiled into the following groups: carbohydrate-binding module, tegument, reticulata-like, lectins, plastoskeletal, and Protein-Targeting-to-STarch (PTST) proteins [27].

Carbohydrate-binding module (CBM) proteins were detected in the putative plastoskeleton samples. CBMs are found in a broad range of proteins that interact with carbohydrates and do not impart enzymatic activity. CBMs are still found in enzymatic proteins such as glycoside hydrolases, polysaccharide lyases, polysaccharide oxidases, glycosyltransferases, expansins, and lectins [28]. Of all the unknown/uncharacterized proteins found in this study, two feature CBMs that have been confirmed by CAZy domain analysis. Both were found in the aqueous distal fraction of dispersion-prepared starch granules: FLOURY6 (Q10F03), which has a CBMF48 domain spanning 100 residues and is crucial for glycogen binding, and an uncharacterized protein (Q6YXZ6) featuring an X8/CBMF43 domain.

Protein Q6YXZ6 has been annotated as glucan endo-1,3-beta-glucosidase 6 in the NCBI database and contains a CBMF43 or X8 carbohydrate-binding domain. These domains are 90–100 residues in length and bind β-1,3-glucans [29]. CBMF43 domains are also present in structural support proteins in *A. thaliana* [30].

Lectins, reticulata related-like, tegument, structural, and PTST proteins were observed in the putative plastoskeleton samples. A small group of putative SLS-related proteins were identified in the proteomes of dispersion-prepared granules (Table 3). The microsieve and flotation starch preparation methods did not reveal any putative SGAP architecture proteins. Two proteins with tegument domains were found in the distal proteome: transport protein sec24-like (Q0JF82) and altered inheritance of mitochondria protein 3 (Q5JML5). Transport protein sec24-like was also present in the amylome. Two proteins with reticulata related-like domains were found in the distal proteome (RER4; Q5JK51) and amylome (RER3; Q5VQR0). A single lectin protein (Ricin B-like lectin R40C1-domain containing protein (Q10M12)) was found in the distal proteome of dispersion-prepared granules. Lectins bind galactose in other organisms [31,32], but CAZy did not identify any known CBMs in this plant homolog. Eight structural proteins were found in the distal fraction and were classified by the NCBI Batch CD database based on the presence of the WD-40 domain (Q0D3Z9, Q9AWU6, Q2QX21), the scaffolding domain SPFH-prohibitin (Q7EYR6), and the PH-like superfamily domain found in proteins involved in membrane curvature (Q654U5). PantherDB attributed the following functions to the same group: vesicle coat protein (Q0D3Z9), microtubule family cytoskeletal protein (Q654U5), non-motor actin-binding protein (Q9AWU6), microtubule-binding motor protein (Q5N7E8), myelin protein (Q6ZIG6), and microtubule-binding protein (Q5NBL8). A single PTST-related protein (Q6Z0Y8) presented in the proximal amylome of dispersion-extracted starch granules, but no CBMs were identified in this peptide using the CAZy database.

## 3. Discussion

In the rice kernel, starch is the major storage carbohydrate and this metabolic reserve supplies the germinating embryo with an energy source. These molecules accumulate to high levels in the cereal endosperm and have evolved to be packaged efficiently within the cell [33]. In *Oryza*, this packaging takes the form of the compound granule, an intra-organellar anatomical feature also present in *Avena*.

Proteins were extracted from rice starch granules prepared using three different methods, as follows:

1. Dispersion-based disruption of the compound granule using osmotic buffer;
2. Microsieving;
3. Flotation-based disruption using a cesium-chloride gradient.

The dispersion preparation method obtained the highest number of unique SGAPs; conversely, flotation preparation obtained the lowest number of unique SGAPs. The top hits in each dataset were primarily seed storage and biosynthesis proteins, although the dispersion method isolated a wide variety of structural proteins in both the distal proteome and proximal amylome.

Known starch granule-associated proteins (SGAPs) were detected by mass spectrometry [34]. The starch granule proteome of commercially prepared rice starch has already been examined using trypsinization and isopropanol solubilization [16]. The mentioned study outlined the rice starch granule proteome and examined the population of SGAP starch biosynthesis, metabolism, and seed storage proteins. However, since the reference material was commercially processed, most of the SGAP protein may have been removed during processing. Protein remaining on the starch granule after processing affects the quality and final yield of the pure starch remaining; because the study utilized commercially processed starch, it was feasible that some SGAPs had already been removed prior to analysis. In the current study, multiple starch granule preparation methods were used on native kernels to obtain a more accurate representation of the rice starch granule proteome.

Trypsin treatments were performed on these starch samples and the water-soluble peptides liberated into the supernatant were sequenced. The remaining hydrophobic proteins were released from the starch granule surface by isopropanol solubilization. These protein extraction methods isolated primarily carbohydrate metabolism and seed storage proteins, as expected [15]. This comprehensive list includes globulins, glutelins, prolamins, and other typical SGAPs such as starch biosynthetic enzymes, starch mobilization enzymes, heat shock proteins (required for normal amyloplast development) [35], and 14-3-3 proteins (required for the assembly of starch biosynthetic complexes) [36], as well as putative compound granule framework proteins (Datasets S1–S6).

All six datasets feature the characteristic starch metabolism, biosynthesis, and storage proteins. The content is affected by the starch preparation method used, as seed proteins have varying solubilities [37] and, as such, are divided into four solubility classes (the Osborne fractions): water-soluble albumins, water-insoluble globulins, alcohol-soluble gliadins, and insoluble glutenins [38]. As the first step of each method used imbibed and wet-ground rice, the albumins were solubilized and discarded early in the starch granule preparation. The use of low-salt buffers and alcohol washes in all three methods removed most of the globulins and gliadins, respectively. The methods used in this study allowed glutelins to remain on the starch granules after pelleting and air drying prior to protein extraction, as glutelins represent a major fraction of each rice grain proteome [39,40].

Eleven peptides were common to the aqueous supernatants obtained from all three starch preparation methods and twenty-eight were common to the isopropanol-solubilized fraction obtained from all three methods. We found that the core proteomes are relatively sparse (Table 2) considering that rice has 50,000 genes [41]. The majority of the remaining proteins are seed storage and starch-metabolism-related (glutelins, sucrose synthase enzymes, starch-branching enzymes), as expected [16].

Notably, Brittle1 (BT1) [6] was identified in all six datasets collected, with the exception of the isopropanol-solubilized fraction from the flotation-prepared starch granules. However, since SDS can be used to extract loosely bound proteins from the granule [42], the majority of proteins are likely removed and discarded during granule preparation, which is reflected in the comparatively shorter list of uniquely identified proteins associated with flotation-prepared starch granules. However, these data are still valuable as they indicate which proteins remain bound to the starch granule following a relatively destructive preparation method.

**Figure 3:**
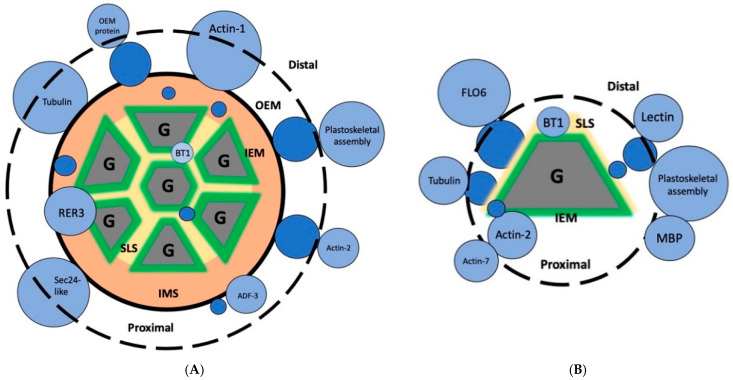
Distal proteomes and proximal amylomes of the rice starch granule represented as schematic sets. (**A**) An intact starch grain. (**B**) An isolated starch granule (G). ADF, actin-depolymerizing factor. BT1, Brittle-1. IEM, inner envelope membrane (green). IMS, intermembrane space (orange). MBP, microtubule-binding protein. OEM, outer envelope membrane (black). RER, reticulata-related. SLS, septum-like structure (yellow). Known and hypothetical starch grain framework proteins are in blue [4,5,6]. Proteins not drawn to scale.

The three methods used have shown that diverse starch granule extraction methods and proteomics analysis techniques can help to map the putative plastoskeleton of the rice SG. A hypothetical schema of the rice SG and granule has been proposed (Figure 3). Both intact grains (Figure 3A) and individual granules (Figure 3B) contributed to the SGAP proteomes analyzed in this study. Most of the peptide hits were found in the dispersion-prepared starch granule extracts, primarily in the aqueous fraction (Table 3). Actin-de-polymerizing factors (ADFs) are also present in the distal proteome and the amylome of the starch granule (Table 2). The presence of a microtubule family cytoskeletal protein with a putative role in membrane curvature (Q654U5) could be a significant actor in actin-related plastoskeletal formation [43].

The presence of plastid-related proteins identified by domain homology (reticulata and tegument) may also be a key towards elucidating compound starch granule organization. It was reported that the reticulata-related (rer) gene family in *Arabidopsis thaliana* is involved in chloroplast formation and presents a reticulated phenotype in plant leaves [44]. RER proteins localize to the outer and inner envelope membranes of the chloroplast [45]. This study identified rice homologs of the *A. thaliana* proteins reticulata-related 3 (RER3, also known as alpha-tandem) and reticulata-related 4 (RER4, also known as MEP3) in the distal proteome of the starch granule. As with their *A. thaliana* homologs, these proteins feature domains of unknown function [46]. Since the amyloplast is structurally and developmentally analogous to the chloroplast [47], it is hypothesized that rice RER3 and RER4 also localize to the amyloplast envelope membranes (Figure 3A). Putative SGAP Ricin B-like lectin R40C1-domain containing peptide is presented here as an SLS component (Figure 3B) due to its predicted cementitious nature [32]. The tegument group protein altered inheritance of mitochondria protein 3 has putative actin patch activity; in yeast, actin patch proteins form localized structural patches, which play a role in budding and fission by constricting the cell membrane [48]. It is hypothesized that a similar mechanism may occur in the later stages of endosperm development, by which amyloplasts are proposed to generate new amyloplasts via a budding-type mechanism [6].

Gleaned from these analyses, the data present many interesting avenues of future rice SGAP studies, in addition to investigating the SGAP using a plastoskeletal-focused approach: E3 ubiquitin ligases, as one example, were discovered in the distal proteome and so may play a pivotal role in the development of plastid components. There is a paucity of data regarding ubiquitination and 26S proteasome involvement in plastid development, and even less with amyloplasts. The presence of the E3 ligases begs the question of whether there is a mechanism within the amyloplast to prevent the translocation of specific proteins into the amyloplast [49]. Similarly, the presence of ENOD93 (early nodulin-93) in the amylome may be a point of interest—early nodulins have been identified as candidates in quantitative trait loci analyses as being associated with starch quality traits such as glassiness and chalkiness [50]. It would be worth examining further the role of ENOD93 in amyloplast development and whether such development can be linked to the quality of the rice grain.

This study revealed an extensive SGAP network, and that the outcome of proteomic analysis can differ significantly as a function of the methodology used. However, there were two major limitations that should be addressed for future SG experiments:

1. This analysis used mature rice kernels, and so limits analysis on SGAPs involved in grain architecture during development (such as plastid division proteins), which will no longer be present in the mature endosperm. A time-course analysis of the SGAP proteome during rice kernel development must be performed to obtain a dynamic model. Observing the development of a simple SG, such as in maize, would provide side-by-side proteomic comparisons and could reveal novel candidates for compound SG architecture development. Whether these types of organization differ in starch mobilization rate is unknown and should also be investigated.
2. Each method of preparation disrupts SGs into individual granules (Figure 2), suggesting that the internal or core SLS proteins are as exposed to the protein extraction methods as the proteins in the distal proteome. A fine-tuned, gentler approach would involve the preparation of intact SGs so that one can distinguish between the outer and inner proteome of the rice starch grain.

Furthermore, this study also establishes the groundwork for the functional characterization of putative plastoskeletal candidates and other SGAPs. The development of overexpression and RNAi-knockdown plant lines can assess the impact of these candidates on amyloplast development and SG formation.

## 4. Materials and Methods

### 4.1. Plant Material

Rice kernels (*Oryza sativa* L. ssp. *japonica* cv. Nipponbare) were obtained from the USDA (Genetic Stocks—Oryza (GSOR) Collection, Stuttgart, AR, US). Kernels were de-husked manually and soaked overnight in sterile double-distilled water (18 h) at 4 °C and then de-germed prior to surface preparation.

### 4.2. Starch Granule Preparation

Three distinct preparation methods were selected to disrupt the rice kernel into individual subunits (granules). Methods varied in type of physical disruption, pH, osmotic potential, and detergent use.

1. Dispersion method [51]: rice kernels (5 g) were ground via mortar and pestle for five minutes prior to the addition of 10 mL starch extraction buffer (50 mM Tris-HCl, pH 7; 10% glycerol; 10 mM EDTA; 1.25 mM DTT). The sample was subjected to vacuum sieve filtration through a 106 μm sieve and the resulting filtrate was centrifuged (4600× *g* for 15 min at 4 °C). The supernatant was discarded, and the pellet was resuspended with 5 mL starch extraction buffer. The dispersion was subjected to vacuum sieve filtration through a 20 μm sieve. The filtrate was washed with starch extraction buffer followed by cold 95% ethanol and acetone. Centrifuging was performed between each wash (8000× *g*, 10 min, 4 °C). Pellets were air-dried under laminar flow for 48 h.
2. Microsieve method [52]: rice kernels (5 g) were manually ground via mortar and pestle for five minutes. Then, 10 mL of sterile double-distilled water was added before continuing the grinding process for an additional five minutes. This slurry was filtered through five layers of cheesecloth and reground for two minutes with mortar and pestle. The resulting dispersion was transferred to a vacuum sieve and filtered through 106 μm, 53 μm, and 20 μm sieves (Gilson Company, Inc., Lewis Center, OH, USA) in series. The filtrate was centrifuged twice (4600× *g* for 15 min at 4 °C) and the supernatant was discarded. Pellets were air-dried under laminar flow for 48 h.
3. Flotation method [53]: rice kernels (5 g) were manually ground via mortar and pestle for five minutes. Then, 10 mL of sterile double-distilled water was added before continuing the grinding process for an additional five minutes. The dispersion was filtered through five layers of cheesecloth and centrifuged (4600× *g* for 15 min at 4 °C). The supernatant was discarded, and the pellet was resuspended in 1 mL of sterile double-distilled water overlaid with 80% *w*/*v* cesium chloride. The solution was centrifuged (4600× *g* for five minutes at 4 °C) and the supernatant discarded. The pellet was washed with a wash buffer (62.5 mM Tris-HCl, pH 6.8; 10 mM EDTA; 4% SDS), sterile double-distilled water, and acetone. Centrifuging was performed between each wash (8000× *g* at 10 min for 4 °C). Pellets were air-dried under laminar flow for 48 h.

### 4.3. Peptide Preparation

The peptides associated with the starch granule surface were collected according to a modified protocol [15,17]. Trypsin-treated granules were centrifuged at 18,000× *g* for one minute and supernatant was transferred to a fresh tube. Pellets were washed five times with a 10-fold excess of double-distilled H_2_O to remove residual water-soluble proteins. Following water washing, proteins remaining on the starch granule surface were extracted by adding 350 μL of 50% (*v*/*v*) isopropanol and 50 mM NaCl and gently agitated for 45 min at room temperature. The samples were centrifuged at 18,000× *g* for one minute, and the supernatant was collected. The peptides from both this isopropanol fraction and the previously reserved aqueous supernatant were dried in a Speed Vac (Speed Vac Concentrator model SVC 100H; Savant Instruments Inc., Hicksville, NY, USA). Peptide pellets were resuspended in 40 μL of double-distilled H_2_O, purified using ZipTips with C18 resin (MilliporeSigma, Bedford, MA, USA) to remove salt and residual starch, and dried in a Speed Vac. Peptides were resuspended in 40 μL of 0.1% formic acid.

### 4.4. Scanning Electron Microscopy (SEM)

Starch samples obtained from each method were mounted on aluminum stubs and subjected to pressurized air under a vacuum hood to produce a thin layer. Stubs were sputter-coated with gold (Gatan Model 882 PECS) and analyzed by SEM (JSM-7500F FESEM, Materials Characterization Facility, University of Ottawa, ON, Canada).

### 4.5. Nano-HPLC-MS/MS Analyses of Peptides

Aliquots of dry rice starch powders were incubated with proteomics-grade trypsin (Sigma-Aldrich, St. Louis, MO, USA) at a ratio of 24 μL:1 mg at 37 °C for 18 h with gentle agitation. The supernatant was collected and analyzed by LC-MS/MS using the following protocol [54]. Further aliquots were incubated with 50% isopropanol:50 mM NaCl solution for 45 min at room temperature with gentle agitation. The supernatant was collected and analyzed by LC-MS/MS. Samples were re-suspended with 25 μL of 1% FA in water and 2 μL was injected into the LC/MS/MS. All experiments were performed on an Orbitrap Fusion (Thermo Fisher Scientific, Waltham, MA, USA) coupled to an UltiMate 3000 nanoRLSC (Dionex, Sunnyvale, CA, USA). Peptides were separated on an in-house packed column (Polymicro Technology, Phoenix, AZ, USA), 15 cm × 70 μm ID, Luna C18(2), 3 μm, 100 Å (Phenomenex, Torrance, CA, US), employing a water/acetonitrile/0.1% formic acid gradient. Samples were loaded onto the column for 105 min at a flow rate of 0.30 μL/min. Peptides were separated using 2% acetonitrile in the first 7 min and then using a linear gradient from 2 to 38% of acetonitrile for 70 min, followed by gradient from 38 to 98% of acetonitrile for 9 min, then at 98% of acetonitrile for 10 min, followed by gradient from 98 to 2% of acetonitrile for 3 min and wash 10 min at 2% of acetonitrile. Eluted peptides were directly sprayed into the mass spectrometer using positive electrospray ionization (ESI) at an ion source temperature of 250 °C and an ion spray voltage of 2.1 kV. Full-scan MS spectra (*m*/*z* 350–2000) were acquired at a resolution of 60,000. Precursor ions were filtered according to monoisotopic precursor selection, charge state (+2 to + 7), and dynamic exclusion 30 s. The automatic gain control settings were 4 × 10^5^ for full FTMS scans and 1 × 10^4^ for MS/MS scans. Fragmentation was performed with collision-induced dissociation (CID) in the linear ion trap. Precursors were isolated using a 2 *m*/*z* isolation window and fragmented with a normalized collision energy of 35%.

### 4.6. Peptide Identification

Proteome Discoverer 2.1 (Thermo Fisher Scientific, Waltham, MA, USA) was used for peptide identification [55]. The precursor mass tolerance was set at 10 ppm and 0.6 Da mass tolerance for fragment ions. Search engine SEQUEST-HT, implemented in Proteome Discovery [56], was applied for all MS raw files. Search parameters were set to allow for dynamic modification of methionine oxidation, acetyl on N-terminus, and static modification of cysteine carbamidomethylation. The search database consisted of nonredundant/reviewed *Oryza sativa* ssp. japonica protein sequences in FASTA file format from the UniProt/SwissProt database [24]. The False Discovery Rate (FDR) was set to 0.05 for both peptide and protein identifications.

### 4.7. Bioinformatics Analysis

Peptides with High Protein FDR ranking were selected from the mass spectrometric data for further analysis. All peptides were ranked according to SEQUEST score. Bioinformatics analysis was performed using the NCBI Web CD-Search Tool (www.ncbi.nlm.nih.gov/Structure/bwrpsb/bwrpsb.cgi accessed on 4 October 2019) of the NCBI Batch Conserved domain database [19] (updated 26 March 2020) to identify structural domains in unknown proteins [25]. InteractiVenn was used to find commonalities between proteomes [21]. UniProt Retrieve/ID mapping (www.uniprot.org/uploadlists/ accessed on 4 October 2019) [24], CAZy (www.cazy.org accessed on 4 October 2019) [26], and the PantherDB v14.1 Gene List analysis (pantherdb.org accessed on 4 October 2019) [20] were used to further characterize unknown proteins. Functional enrichment analysis was performed using Gene Ontology (GO) Enrichment Analysis powered by Panther (geneontology.org accessed on 4 October 2019) [20].

### 4.8. Public Database Repository

The mass spectrometry proteomics data have been deposited in the ProteomeXchange Consortium via the PRIDE [57] partner repository with the dataset identifier PXD032314.

## Figures and Tables

**Table 1:** Number of peptides identified by mass spectrometric analysis.

Starch Preparation Method	Protein Extraction Method	# of Peptides (# Uncharacterized)
Dispersion	Trypsin	1575(663)
	Isopropanol	559(212)
Microsieving	Trypsin	123(39)
	Isopropanol	154(45)
Flotation	Trypsin	24(8)
	Isopropanol	49(10)

**Table 2:** Common proteins in the distal proteome and proximal amylome.

UniProt ID	Description
**Carbohydrate Synthesis and Metabolism**	
**Q0JKM8**	**Aspartic proteinase oryzasin-1-like**
**Q852L2**	**Cupincin**
B7EVB8	Glucose-1-phosphate adenylyltransferase
P15280	Glucose-1-phosphate adenylyltransferase small subunit 2 *
**Q65XK0**	**Ketol-acid reductoisomerase**
**Q75M03**	Putative H+-pyrophosphatase
Q6AVA8	Pyruvate, phosphate dikinase 1, chloroplastic *
Q6ZBH2	Sorbitol dehydrogenase *
B3VDJ4	Starch branching enzyme
**Q43009**	**Sucrose synthase 3**
**Q93X08**	**UTP-glucose-1-phosphate uridylyltransferase**
**Miscellaneous**	
**Q6ZKC0**	**14-3-3-like protein GF14-C**
Q6Z782	Brittle1 (BT1)
**Q0DEP9**	**Early nodulin-93**
**A0A0P0WFP9**	**Fatty acid export 2, chloroplastic**
**Q6ZHP6**	**Outer envelope membrane protein 7**
**Q9FWV6**	**Probable aquaporin TIP3-1**
**Q10Q18**	**Transport protein Sec61 alpha subunit isoform 2**
**Q6Z0Z9**	**V-type proton ATPase proteolipid subunit**
**Seed Reserve**	
Q8GVK5	13 kDa prolamin
Q75GX9	63 kDa globulin-like protein
Q6K7K6	Glutelin
A1YQG5	Glutelin
**Q6ESW6**	**Glutelin**
**P07728**	**Glutelin type-A 1**
P07730	Glutelin type-A 2
**Q09151**	**Glutelin type-A 3**
**P14323**	**Glutelin type-B 1**
**Q02897**	**Glutelin type-B 2**
**Q8S0E1**	**Patatin**
**Q65XA1**	**Putative legumin**
**Q0DS36**	**Vicilin-like seed storage protein**
**Stress Response**	
**Q8H920**	**AWPM-19-like family protein**
**Q6Z7B0**	**Heat shock 70 kDa protein BIP1**
Q75LL0	Putative stress-related protein
**Transcriptional/Translational Machinery**	
**O64937**	**Elongation factor 1-alpha**
**Q6L500**	**Probable histone H2A.4**
**Q2QS71**	**Probable histone H2A.7**

Bold entries were identified in the isopropanol-solubilized peptide fraction. * Common to all datasets.

**Table 3:** Plastoskeletal proteins from dispersion-method-prepared starch granules.

UniProt ID	Description	Score	Coverage (%) ^a^	# Peptides ^b^
**Actin**				
Q10DV7	Actin-1	85.957	51.1936	15(3)
Q75HX0	Actin-1	79.968	44.9468	12(9)
A3C6D7	Actin-2	77.034	50.9284	14(3)
Q67G20	**Actin-7**	71.587	46.6843	13(1)
Q10DV7	**Actin-1**	27.620	38.1963	9(1)
Q75HX0	**Actin-1**	20.401	22.3404	6(4)
P0C540	**Actin-7**	14.061	21.0106	6(1)
Q9AY76	Actin-depolymerizing factor 2	13.516	22.0690	2(2)
Q84TB6	Actin-depolymerizing factor 3	6.3583	34.6667	3(3)
Q84TB3	**Actin-depolymerizing factor 4**	4.7321	11.5108	1(1)
Q0DLA3	**Actin-depolymerizing factor 7**	1.5514	8.63309	1(1)
Q84TB6	**Actin-depolymerizing factor 3**	1.5320	9.33333	1(1)
**Lectin**				
Q10M12	Ricin B-like lectin R40C1-domain containing	42.362	49.1379	12(10)
**Membrane-Associated**				
Q75GB3	Outer membrane protein	11.295	6.26896	4(4)
**Structural**				
Q0D3Z9	Transport protein SEC31 homolog B	48.342	14.6406	11(11)
Q7EYR6	Prohibitin-2	32.952	24.9135	5(2)
Q654U5	Phragmoplastin	19.075	7.24479	4(4)
Q9AWU6	WD-repeat containing protein 1	16.142	10.1639	4(4)
Q5N7E8	Microtubule binding motor protein	9.2205	13.3080	3(3)
Q2QX21	Myotonica WD repeat-containing protein	5.8419	4.08526	1(1)
Q6ZIG6	Myosin heavy-chain related protein	2.0988	1.96592	1(1)
Q5NBL8	Klaroid, isoform A-related	1.6375	4.61539	1(1)
**Reticulata Related-Like**				
Q5JK51	Reticulata-related 4-like	1.4744	2.57069	1(1)
Q5VQR0	**Reticulata-related 3-like**	20.840	20.6896	4(4)
**Starch granule-binding**				
Q6YXZ6	Glucan endo-1,3-beta-glucosidase 6	5.8419	4.08526	1(1)
Q10F03	FLOURY6	2.0380	1.51229	1(1)
**Tegument**				
Q0JF82	Transport protein Sec24-like	19.287	7.75946	5(5)
Q0JF82	**Transport protein Sec24-like**	5.5103	4.26770	2(2)
Q5JML5	Altered inheritance of mitochondria protein 3-like	2.6716	2.86195	1(1)
**Tubulin and Tubulin-Like**				
P46265	Tubulin beta-5 chain-like	71.042	32.4385	12(2)
A3ANA0	Tubulin beta-7 chain	70.215	32.6577	12(2)
P45960	Tubulin beta-4 chain-like	66.176	30.4251	11(2)
Q75GI3	Tubulin alpha-1 chain	60.193	40.3548	11(7)
A3AL37	Tubulin beta chain	56.060	24.8918	9(1)
P37832	**Tubulin beta-7 chain**	42.446	30.8559	12(12)
Q0PVB0	Tubulin alpha-1 chain-like	37.643	29.7778	8(4)
Q53M52	**Tubulin alpha-2 chain-like**	18.417	13.5255	4(2)
Q10PW2	Tubulin alpha chain, putative	15.823	22.4944	6(4)
P28752	**Tubulin alpha-1 chain**	15.433	13.5556	4(2)
Q10PW2	**Tubulin alpha chain, putative**	3.8827	3.11804	1(1)

^a^ Percent protein sequence coverage by total peptides. ^b^ Number of total peptides (number of unique peptides). Bold entries were sequenced from the isopropanol-solubilized peptide fraction.

**Table 4:** Plastoskeletal proteins from microsieve-prepared starch granules (isopropanol fraction).

UniProt ID	Description	Score	Coverage (%) ^a^	# Peptides ^b^
**Actin**				
Q10DV7	Actin-1	27.619	38.1962	1(1)
Q10AZ4	Actin-3	1.2836	6.10079	1(1)
**Tubulin**				
Q53M52	Tubulin alpha-2 chain	1.5231	3.32594	1(1)
P45960	Tubulin beta-4 chain	1.5136	8.50111	2(2)

^a^ Percent protein sequence coverage by total peptides. ^b^ Number of total peptides (number of unique peptides).

## Data Availability

Section 4.8 above is new and covers public archiving accessibility—4.8. Public Database Repository.

## Supplementary Materials

The following are available online: https://www.mdpi.com/article/10.3390/molecules27103307/s1, Figure S1. Common proteomes. (A) The common distal proteome represents proteins identified following trypsin-shaving of the starch granules (left). The common proximal amylome represents proteins found in all three supernatants following isopropanol-solubilization of residual peptides (right). Figures generated using InteractiVenn (interactivenn.net) (Heberle et al., 2015). (B) Common proteins in the core distal proteome and core proximal amylome; Figure S2 Common proteomes of starch granules prepared by dispersion, microsieve, and flotation methods. (Total peptides). Figure generated using InteractiVenn (interactivenn.net) (Heberle et al., 2015); Figure S3. Gene ontology (GO) categorization of uncharacterized starch granule-associated proteins (SGAPs) extracted from dispersion-method prepared granules. Bar charts generated using Gene Ontology (GO) Enrichment Analysis powered by Panther (Mi et al., 2018). BP, biological process. MF, molecular function. CC, cellular compartment; Figure S4. Gene ontology (GO) categorization of uncharacterized starch granule-associated proteins (SGAPs) extracted from microsieve-prepared granules. Bar charts generated using Gene Ontology (GO) Enrichment Analysis powered by Panther (Mi et al., 2018). BP, biological process. MF, molecular function. CC, cellular compartment; Figure S5. Gene ontology (GO) categorization of uncharacterized starch granule-associated proteins (SGAPs) extracted from flotation-prepared granules. Bar charts generated using Gene Ontology (GO) Enrichment Analysis powered by Panther (Mi et al., 2018). BP, biological process. MF, molecular function. CC, cellular compartment; Table S1. Uncharacterized proteins in the distal proteome of dispersion-prepared granules; Table S2. Uncharacterized proteins in the proximal amylome of dispersion method-prepared starch granules; Table S3 Uncharacterized proteins in the distal proteome of microsieve-prepared starch granules; Table S4 Uncharacterized proteins in the proximal amylome of microsieve-prepared starch granules; Table S5 Uncharacterized proteins in the distal proteome of flotation-prepared starch granules; Table S6 Uncharacterized proteins in the proximal amylome of flotation-prepared starch granules; Dataset S1. Total trypsin-shaved proteome from dispersion method-prepared granules; Dataset S2. Total trypsin-shaved proteome from microsieve method-prepared granules; Dataset S3. Total trypsin-shaved proteome from flotation method-prepared granules; Dataset S4. Total isopropanol-solubilized proteome from dispersion method-prepared granules; Dataset S5. Total isopropanol-solubilized proteome from microsieve method-prepared granules; Dataset S6. Total isopropanol-solubilized proteome from flotation method-prepared granules; Dataset S7. Peptides unique to the trypsin-shaved proteome from dispersion method-prepared granules; Dataset S8. Peptides unique to the trypsin-shaved proteome from microsieve method-prepared granules; Dataset S9. Peptides unique to the trypsin-shaved proteome from flotation method-prepared granules; Dataset S10. Peptides unique to the isopropanol-solubilized proteome from dispersion method-prepared granules; Dataset S11. Peptides unique to the isopropanol-solubilized proteome from microsieve method-prepared granules; Dataset S12. Peptides unique to the isopropanol-solubilized proteome from flotation method-prepared granules.

## Sample Availability

Samples of the compounds are not available from the authors.
