# Supplementary material for: Three Diverse Granule Preparation Methods for Proteomic Analysis of Mature Rice (Oryza sativa L.) Starch Grain
Source: Molecules. 2022 May 21;27(10):3307. doi: 10.3390/molecules27103307 (PMC9144640; doi:10.3390/molecules27103307)
Supplement: Supplementary file 1 [file molecules-27-03307-s001.zip › molecules-1538946-supplementary.pdf]

Supplementary Materials file, for article in Molecules (mdpi)

Three Diverse Granule Preparation Methods for Proteomic Analysis of Mature Rice (Oryza sativa L.) Starch Grain

Zachary Provost, Ella Olivia Hansen, Morgan Viola Lynds, Barry S. Flinn, Zoran Minic,
Maxim V. Berezovski and Illimar Altosaar*

***Corresponding author:** Illimar Altosaar Email: altosaar@uottawa.ca Tel: +1 (613) 804-1885

This PDF file includes:

Figures S1 to S5
Tables S1 to S6
Legends for Datasets S1 to S12
SI References

Other supplementary materials include:

Dataset S1
Dataset S2
Dataset S3
Dataset S4
Dataset S5
Dataset S6
Dataset S7
Dataset S8
Dataset S9
Dataset S10
Dataset S11
Dataset S12

A

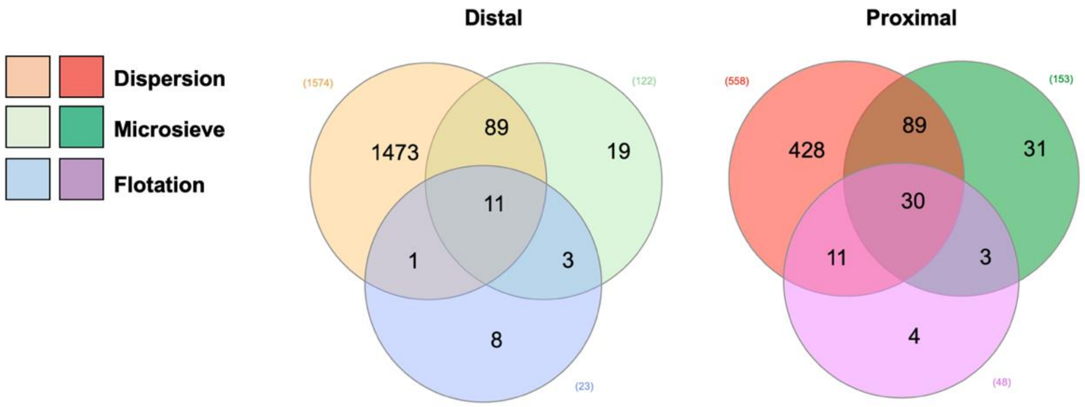

B

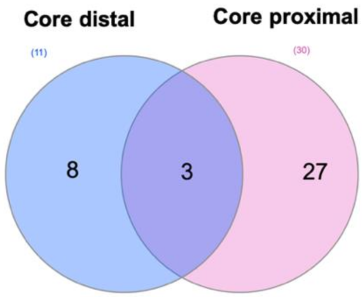

Fig. S1 Common proteomes. **A** The common distal proteome represents proteins identified following trypsin-shaving of the starch granules (left). The common proximal amylome represents proteins found in all three supernatants following isopropanol-solubilization of residual peptides (right). Figures generated using InteractiVenn (interactivenn.net) (Heberle et al. 2015). **B** Common proteins in the core distal proteome and core proximal amylome

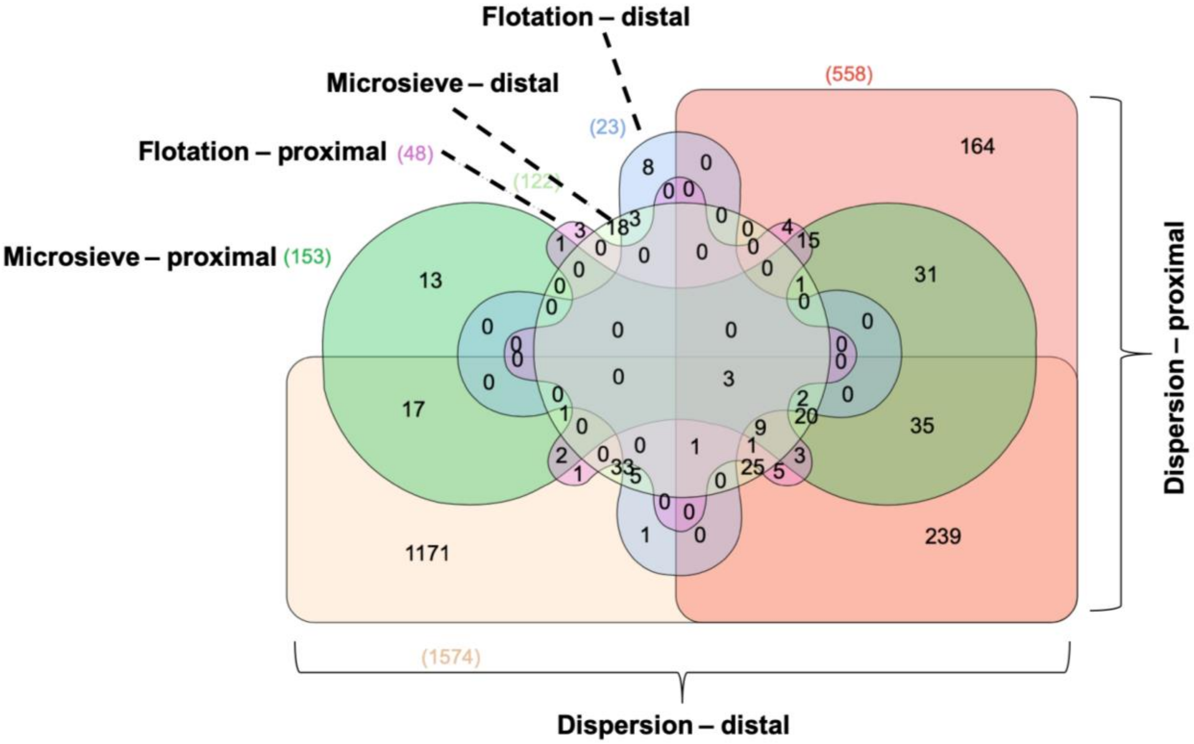

Fig. S2 Common proteomes of starch granules prepared by dispersion, microsieve, and flotation methods. (Total peptides). Figure generated using InteractiVenn (interactivenn.net) (Heberle et al. 2015)

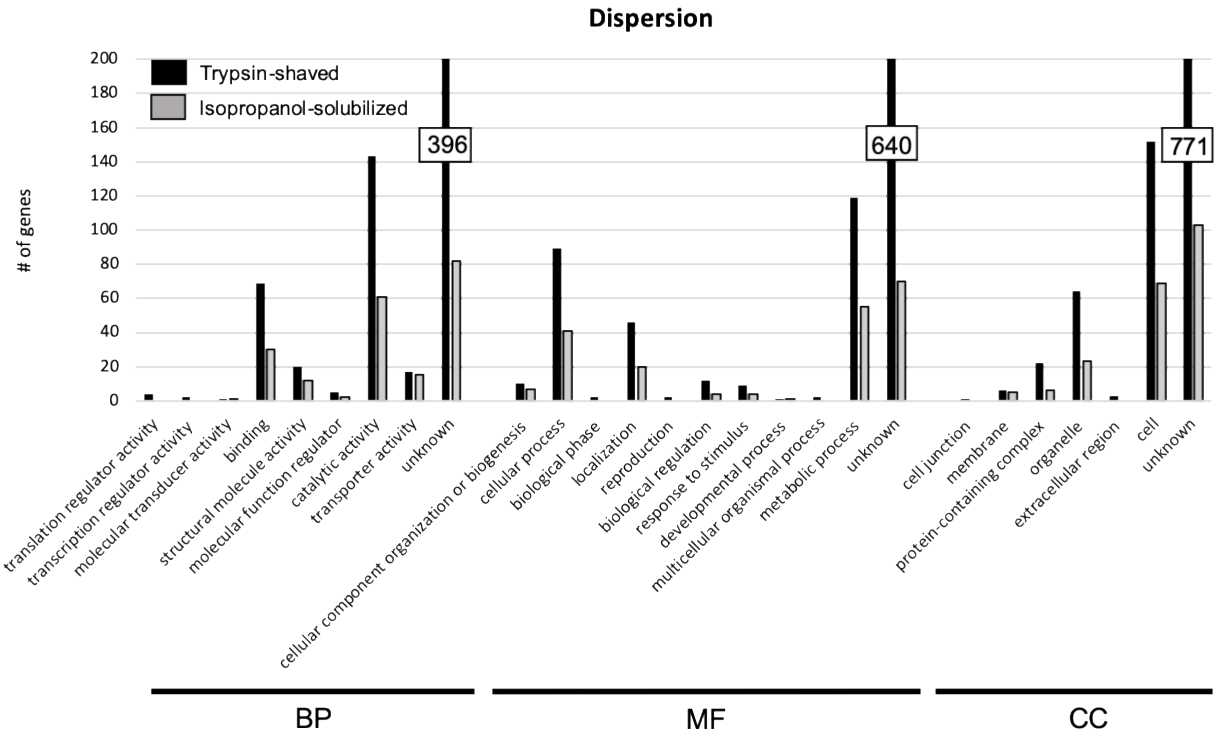

Fig. S3 Gene ontology (GO) categorization of uncharacterized starch granule-associated proteins (SGAPs) extracted from dispersion-method prepared granules. Bar charts generated using Gene Ontology (GO) Enrichment Analysis powered by Panther (Mi et al. 2018). BP, biological process. MF, molecular function. CC, cellular compartment

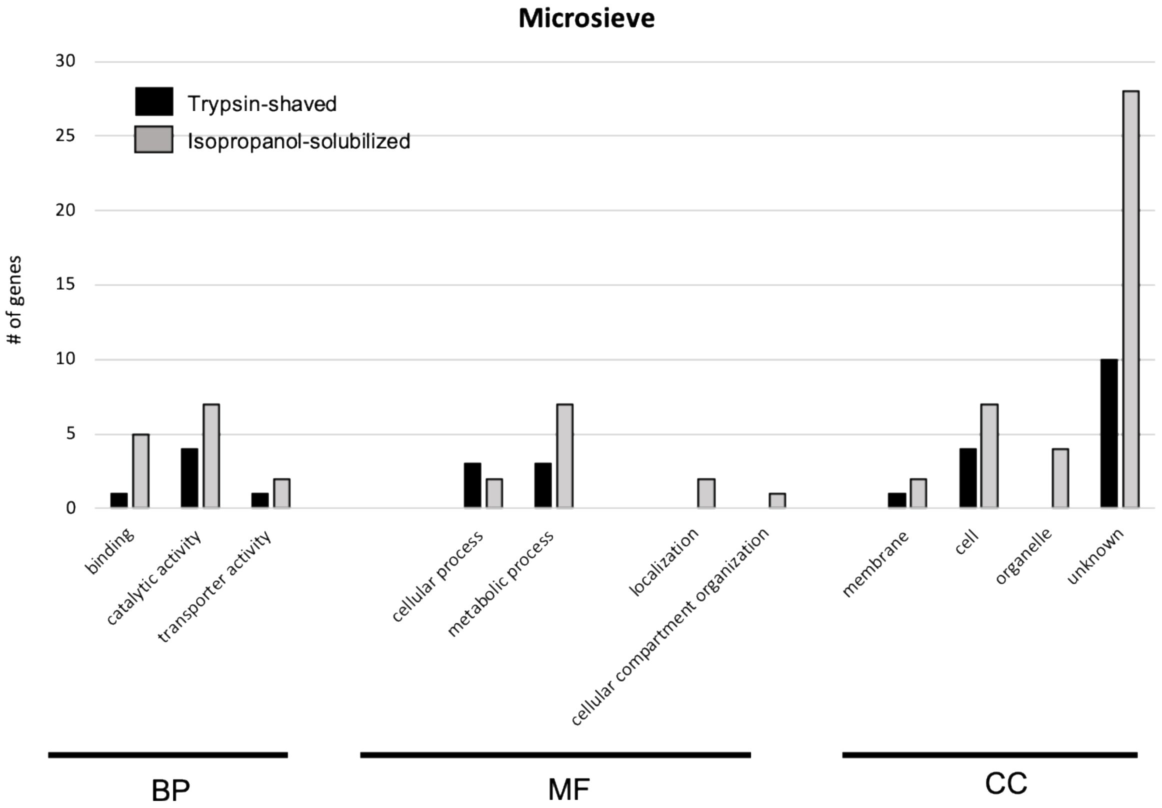

Fig. S4 Gene ontology (GO) categorization of uncharacterized starch granule-associated proteins (SGAPs) extracted from microsieve-prepared granules. Bar charts generated using Gene Ontology (GO) Enrichment Analysis powered by Panther (Mi et al. 2018). BP, biological process. MF, molecular function. CC, cellular compartment

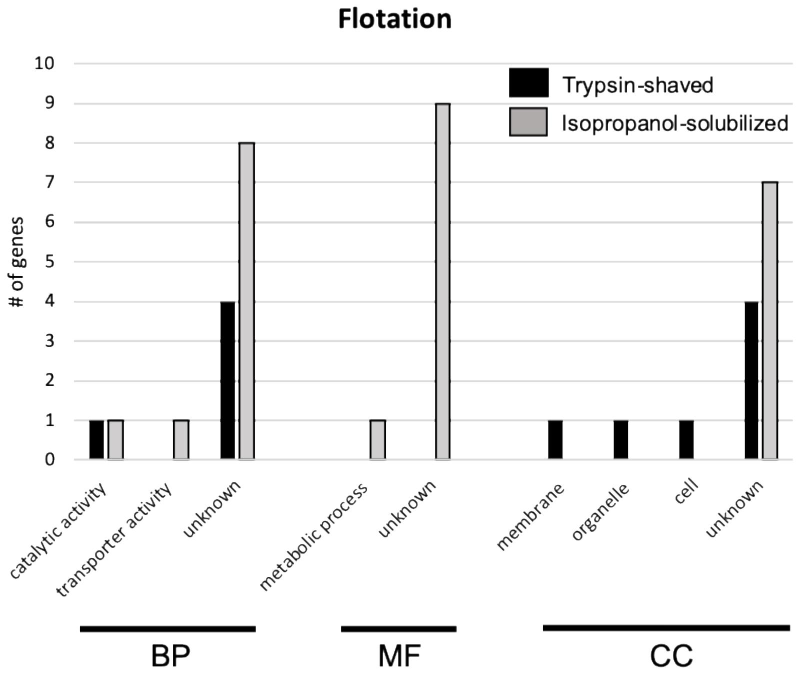

Fig. S5 Gene ontology (GO) categorization of uncharacterized starch granule-associated proteins (SGAPs) extracted from flotation-prepared granules. Bar charts generated using Gene Ontology (GO) Enrichment Analysis powered by Panther (Mi et al. 2018). BP, biological process. MF, molecular function. CC, cellular compartment

Table S1 Uncharacterized proteins in the distal proteome of dispersion-prepared granules

UniProt ID	Description	Score	Coverage (%)[a]	# peptides[b]
**Amino acid biosynthesis**				
Q5N9Z8	Bifunctional aminotransferase	31.092	28.3843	7(7)
Q8LRJ4	Peptide-N4-(N-acetyl-beta-glucosaminyl) asparagine amidase	7.2003	4.49438	2(2)
Q5NBQ1	Histidine biosynthesis bifunctional protein	6.6319	6.53595	1(1)
Q9FTU2	Replication protein A, class 2b aminoacyl-tRNA synthetase	5.7891	26.9503	3(3)
Q8LR75	Triosephosphate isomerase	3.9846	12.5490	1(1)
Q5N7G9	Membrane trafficking regulatory protein	1.4680	3.00752	1(1)
**Biosynthesis of secondary metabolites**				
Q5N7Y9	Hydroxyphenylpyruvate reductase	4.4002	7.91139	1(1)
**Carbohydrate metabolism**				
Q0JKM8	Aspartic proteinase oryzasin-1-like	75.502	27.2031	11(11)
Q9AWZ5	Late embryogenesis abundant protein Lea14-A	22.648	49.0066	5(5)
**Light response**				
Q0JNL8	Cop9 signalosome complex subunit 2	6.2640	6.15034	2(2)
**Lipid metabolism**				
Q5SNJ4	Metalloprotease	32.130	27.5247	7(7)
Q94E74	Adenine nucleotide alpha hydrolases-like	19.974	54.4910	4(4)
Q9FTG9	Acyl transferase 9-like	7.6716	5.89623	2(2)
Q5ZC86	Glutathione S-transferase 4	7.3226	13.3333	2(2)
Q5QM39	Cinnamoyl-CoA reductase 1	3.1384	3.57143	1(1)
**Membrane-associated**				
Q0JQX6	Plastidic glucose transporter 4	41.588	19.7417	7(7)
Q9LGA3	Transmembrane protein 214	30.311	22.1843	8(8)
Q9AS74	Import inner membrane translocase subunit	2.9880	5.52632	1(1)
Q7F5L2	Ectonucleotide pyrophosphatase	1.8008	2.50522	1(1)
Q94E23	Probable receptor-like protein kinase	1.1336	4.26829	1(1)
**Miscellaneous**				
Q9AX15	Probable cysteine desulfurase	9.8278	4.38312	2(2)
Q0JQ41	Kinase	9.7841	10.6267	2(2)
Q5JMl5	Altered inheritance of mitochondria protein 3	2.6716	2.86195	1(1)
**Mitochondrial activity**				
Q9FU74	MAM33 protein	24.130	20.7273	4(4)
**Oxidoreductases**				
Q5ZBH8	Probable aldo-keto reductase 1	6.0404	13.9535	3(3)
Q5SND7	2-oxoglutarate-Fe(II) type oxidoreductase	4.2784	10.1796	2(2)
**Protein trafficking**				
Q9LD54	Envelope ADP, ATP carrier protein, chloroplastic	48.005	31.4961	12(12)
Q9S7H0	Syntaxin-22	3.0910	11.0714	1(1)
**Stress response**				
Q5ZAV7	Universal stress protein PHOS32	20.984	31.2977	4(3)
Q9AWU6	WD repeat-containing protein 1	16.142	10.1639	4(4)
Q8RZ83	Ras-related protein RABA1F	15.138	25.4464	5(3)
Q8S292	Universal stress protein PHOS32	11.999	32.7160	3(3)
Q0JLH6	ATP synthase	3.4440	8.19672	1(1)
Q9FTF3	Rust resistance kinase LR10	1.3689	3.38164	1(1)
**Structural**				
Q5N7Z9	Ras-related protein RAB7	15.134	39.1304	5(3)
Q93W16	GTP-binding protein SAR1A	12.110	27.9793	3(2)
Q5N7E8	Microtubule-binding motor protein	9.2205	13.3080	3(3)
Q5VR46	Leucine-rich repeat extensin-like protein 3	3.1355	3.07692	1(1)
Q8LQJ5	NDR1/HIN1-like protein 6	2.4542	10.6529	1(1)
Q5NBL8	Klaroid, isoform A-related	1.6375	4.61538	1(1)
**Transcriptional/translational machinery**				
Q5VRX8	Glutamine-tRNA ligase	42.734	16.0804	9(8)
Q94E63	Alba chromosomal protein	21.989	38.8158	6(5)
Q93WM3	Asparagine-tRNA ligase, mitochondrial-related	15.599	11.9857	5(5)
Q93VC6	40S ribosomal protein S5	12.038	20.0000	5(5)
Q5NB69	60S acidic ribosomal protein p3-1-related	11.599	22.6891	3(3)
Q5SNH7	60S acidic ribosomal protein P2A	8.6137	28.0702	1(1)
Q5ZBA9	RNA binding protein	5.5209	13.4921	1(1)
A0A0P0V4C6	piRNA biogenesis protein EXD1	3.9738	25.5193	3(3)
Q94EG1	Aromatic aminotransferase ISS1	3.1180	3.55330	1(1)
**Ubiquitin-mediated proteolysis**				
Q8W0I1	Ubiquitin-conjugating enzyme E2 36	16.798	47.7124	5(5)

[a]Percent protein sequence coverage by total peptides.
[b]Number of total peptides (number of unique peptides).

Table S2 Uncharacterized proteins in the proximal amylome of dispersion method-prepared starch granules

UniProt ID	Description	Score	Coverage (%)[a]	# peptides[b]
**Amino acid biosynthesis**				
Q6Z782	Amino acid transporter	83.887	38.8235	15(15)
B9EVR3	Amino acid transporter	14.773	31.3953	3(3)
Q5N9Z8	Bifunctional aspartate aminotransferase	1.0181	3.27511	1(1)
**Carbohydrate metabolism**				
Q0JKM8	Aspartic proteinase oryzasin-1-like	45.878	38.8889	13(13)
Q0JG51	Glycosyl hydrolase family 1	4.0659	12.2302	1(1)
A0A0P0V7G8	CD98 heavy chain, isoform D	1.2423	2.96804	1(1)
**Lipid metabolism**				
Q6Z702	3-isopropylmalate dehydratase large subunit, chloroplastic	17.115	9.14397	3(3)
Q9LGL5	Decarboxylase	5.2303	8.20770	1(1)
Q6ZIH1	Oxygenase	4.4467	4.22265	2(2)
B7F8Y9	Soluble inorganic pyrophosphatase 4	3.4419	7.83410	1(1)
Q0DZN5	Acetolactate synthase small subunit 1, chloroplastic	2.5613	5.58823	1(1)
A0A0P0V2C9	AIG1-like protein, 48352-49494-related	2.0992	0.243355	1(1)
Q5QM65	Fatty acid export 3, chloroplastic	2.0976	5.48780	1(1)
**Membrane-associated**				
Q0JQX6	Plastidic glucose transporter 4	24.960	36.9004	8(8)
Q9LD54	Envelope ADP, ATP carrier protein, chloroplastic-related	13.867	29.6588	8(8)
Q9AWZ5	Putative late embryogenesis-abundant protein	7.0955	25.8278	2(2)
Q0JRG3	Receptor	6.3667	17.5610	2(2)
Q9FP98	Mitochondrial import receptor subunit TOM7	4.4897	21.25	1(1)
Q9LGA3	Transmembrane protein 214	4.0381	3.24232	1(1)
Q6H4V4	Cullin-associated NEDD8-dissociated protein 1	3.5219	2.05254	2(2)
Q6EUQ9	V-type proton ATPase catalytic subunit A	2.3942	2.41546	1(1)
Q6H7E0	Receptor, signaling molecule	1.6178	3.00300	1(1)
Q0DY86	Histidinol-phosphate aminotransferase 1, chloroplastic-related	1.1865	3.76471	1(1)
**Reticulata related-like**				
Q5VQR0	Reticulata-related 2, chloroplastic-related	20.840	20.6897	4(4)
**Starch binding**				
Q6Z0Y8	Protein Targeting to STarch (PTST)-related	2.2595	6.86274	1(1)
**Starch biosynthesis**				
A0A0N7KFE7	1,4-alpha-glucan-branching enzyme	30.778	45.5556	9(9)
**Stress response**				
Q5QMK7	Phosphoglycerate mutase	25.391	19.3202	8(8)
A0A0P0V093	Exportin-2	8.7347	11.2339	1(1)
A0A0P0VLH5	Acetolactate synthase small subunit 1, chloroplastic	6.6611	14.7436	2(2)
Q8RZW7	Methanethiol oxidase	6.2930	10.7884	3(3)
Q5JK10	Aldolase-type TIM barrel family protein	5.9289	5.09259	1(1)
Q6H660	Putative stress-induced protein STI1	4.1221	6.74740	2(2)
Q94E74	Adenine nucleotide alpha hydrolases-like superfamily protein	3.4226	9.58084	1(1)
Q6H734	DNA damage-inducible protein 1	1.9863	3.37349	1(1)
**Transcriptional/translational machinery**				
Q93VC6	40S ribosomal protein s5	18.210	34.5000	5(5)
Q6K1Q6	60S ribosomal protein l14	4.5851	16.4179	2(2)
Q6ETK1	Nucleoid-associated protein YBAB	3.8794	13.5135	2(2)
Q8S292	Adenine nucleotide alpha hydrolase family	2.2070	6.79012	1(1)
Q94E63	Alba chromosomal protein	1.5678	18.4210	1(1)
Q6K667	Ribosomal protein	1.3597	6.50406	1(1)
B9F4A8	28S rRNA (cytosine-c(5))-methyltransferase-related	1.0135	7.72277	1(1)
**Ubiquitin-mediated proteolysis**				
Q8W0I1	Ubiquitin-conjugating enzyme E2 36	4.7198	25.4902	2(2)
**Unknown**				
Q6K2G6	Putative (DUF 3339)-related	20.723	43.4783	1(1)
Q8S9Z3	Unknown	14.350	32.0755	2(2)
Q0DZE5	Unknown	4.7838	4.25532	1(1)
A0A0P0V7T1	Unknown	3.3476	21.1765	1(1)
Q9S7V0	Unknown	3.2783	72.5806	1(1)
A0A0P0UXR0	Unknown	1.0687	19.3833	1(1)

[a]Percent protein sequence coverage by total peptides.
[b]Number of total peptides (number of unique peptides)

Table S3 Uncharacterized proteins in the distal proteome of microsieve-prepared starch granules

UniProt ID	Description	Score	Coverage (%)[a]	# peptides[b]
**Carbohydrate metabolism**				
Q0JQX6	Plastidic glucose transporter 4	10.202	12.1771	2(2)
Q0JKM8	Aspartic proteinase oryzasin-1-like	1.7289	2.87356	1(1)
Q0J1E1	C-1-tetrahydrofolate synthase, cytoplasmic	0.88875	5.94059	1(1)
**Lipid metabolism**				
Q6ZK49	Chloroplast J-like domain 1	0.91793	6.48464	1(1)
**Membrane-associated**				
Q6ZHP6	Outer envelope membrane protein 7	1.5116	18.8119	1(1)
Q67UK4	RING finger, H2 subclass family	0.95078	5.73394	1(1)
**Miscellaneous**				
Q337M4	Vicinal oxygen chelate family	2.7695	8.51648	1(1)
**Oxidoreductases**				
A0A0P0XPG5	Peroxisomal sarcosine oxidase	0.76675	8.17308	1(1)
A0A0P0WTI0	FAD-dependent oxidoreductase family protein	0.64647	3.14607	1(1)
**Pyruvate metabolism**				
Q7XLP7	2,3-dimethylmalate lyase	1.2426	6.16970	1(1)
**Stress response**				
Q67VZ0	PLAT domain-containing protein 2	2.9527	8.77193	1(1)
Q7FAT6	T-complex protein 1 subunit alpha chaperonin	2.1599	3.85321	1(1)
Q6ZJI3	Enzyme modulator	0.65856	1.64609	1(1)
Q6YTX5	Stress-response A/B barrel	0.62672	4.59770	1(1)
**Unknown**				
A0A0P0VBK7	Unknown	1.7326	8.10811	1(1)

24 accession IDs could not be mapped.
[a]Percent protein sequence coverage by total peptides.
[b]Number of total peptides (number of unique peptides).

Table S4 Uncharacterized proteins in the proximal amylome of microsieve-prepared starch granules

UniProt ID	Description	Score	Coverage (%)[a]	# peptides[b]
**Carbohydrate synthesis and metabolism**				
Q0JKM8	Aspartic protease	4.3530	7.47126	2(2)
A0A0N7KFE7	1,4-alpha-glucan-branching enzyme	3.1593	18.8889	2(2)
**Lipid metabolism**				
B7FAF1	Peroxygenase 3-related	2.5156	9.58333	2(2)
C7J0T2	Peroxygenase 1-related	1.8854	3.54839	1(1)
Q7XVN6	Dehydrogenase	1.8502	6.79348	2(2)
**Membrane-associated**				
Q6ZHP6	Outer envelope membrane protein 7	7.9338	31.6832	2(2)
Q0E0Z7	Transmembrane protein	4.0085	44.9275	2(2)
B7F8G3	Translocator protein	3.3189	12.9534	1(1)
Q6ATF8	Membrane traffic protein	2.4483	11.7647	1(1)
Q0DQF2	B12D protein	2.3602	27.0833	1(1)
Q2QM11	Transmembrane protein	2.0786	21.2766	1(1)
B9EVR3	Outer envelope pore protein 16-2	0.82594	11.6279	1(1)
Q6EU87	Transmembrane protein 208	0.80272	9.19540	1(1)
**Miscellaneous**				
Q0DEP9	Early nodulin 93	2.5183	15.1260	1(1)
**Seed reserve**				
Q0DS36	Cupin-like superfamily	3.1558	6.82493	2(2)
Q0DJ38	Prolamin PPROL 14E-like	1.5080	25.4902	2(2)
**Stress response**				
B7EME6	Universal stress protein PHOS32	1.9379	9.52381	2(2)
Q8LQJ5	NDR1/HIN1-like protein 6	0.99140	2.74914	1(1)
Q2R1P5	Disease resistance protein Pik-2-like	0.75399	0.773558	1(1)
**Transcriptional/translational machinery**				
Q0JAC6	60S ribosomal protein l7	0.87031	4.09836	1(1)
Q0E4R7	60S ribosomal protein l9-related	0.78094	5.20833	1(1)
**Unknown**				
A0A0P0VUB4	Unknown	22.586	17.4680	7(7)
Q6K2G6	Protein, putative (DUF 3339)-related	9.0261	43.4783	1(1)
Q9S7V0	Unknown	3.5224	54.8387	1(1)
Q8S9Z3	Unknown	1.4412	14.1509	1(1)
Q8S2H7	Unknown	1.07915	9.11854	1(1)
A0A0N7KGK8	Unknown	0.90309	8.97959	1(1)
A0A0P0XY21	Unknown	0.87290	24.8175	1(1)
Q6K313	Unknown	0.61961	6.72269	1(1)

4 accession IDs could not be mapped.

[a]Percent protein sequence coverage by total peptides.

[b]Number of total peptides (number of unique peptides).

Table S5 Uncharacterized proteins in the distal proteome of flotation-prepared starch granules

UniProt ID	Description	Score	Coverage (%)[a]	# peptides[b]
**Miscellaneous**				
Q6Z7F7	F-box protein	0.51456	4.98301	1(1)
**Ubiquitin-mediated proteolysis**				
Q10LG3	Probable E3 ubiquitin-protein ligase	0.53970	5.30035	1(1)
**Unknown**				
Q6ZKA9	Unknown	0.87877	8.47458	1(1)
A0A0P0Y1U1	Unknown	0.56272	62.0690	1(1)

3 accession IDs could not be mapped.
[a]Percent protein sequence coverage by total peptides.
[b]Number of total peptides (number of unique peptides).

Table S6 Uncharacterized proteins in the proximal amylome of flotation-prepared starch granules

UniProt ID	Description	Score	Coverage (%)[a]	# peptides[b]
Carbohydrate metabolism				
Q0JQX6	Plastidic glucose transporter 4	4.9447	12.7306	2(2)
Q0JKM8	Aspartic protease	0.90588	4.59770	1(1)
**Lipid metabolism**				
A0A0P0WFP9	Fatty acid export 2, chloroplastic	0.68782	14.3322	1(1)
**Membrane-associated**				
Q6ZHP6	Outer envelope membrane protein 7	5.1405	25.7426	1(1)
Q9LGA3	Transmembrane protein 214	1.1700	2.38910	1(1)
**Miscellaneous**				
Q0DEP9	Early nodulin 93	1.5173	15.1260	1(1)
**Seed reserve**				
Q0DS36	Cupin-1	1.3606	4.15430	1(1)
**Transcriptional/translational machinery**				
Q7XKI6	Ribosomal RNA small subunit methyltransferase	1.5626	7.98817	1(1)
**Unknown**				
Q7XLP6	Unknown	1.0810	10.9489	1(1)
A0A0P0X2W9	Unknown	0.57900	6.85279	1(1)

[a]Percent protein sequence coverage by the total peptides.
[b]Number of total peptides (number of unique peptides).

Dataset S1 Total trypsin-shaved proteome from dispersion method-prepared granules

Dataset S2 Total trypsin-shaved proteome from microsieve method-prepared granules

Dataset S3 Total trypsin-shaved proteome from flotation method-prepared granules

Dataset S4 Total isopropanol-solubilized proteome from dispersion method-prepared granules

Dataset S5 Total isopropanol-solubilized proteome from microsieve method-prepared granules

Dataset S6 Total isopropanol-solubilized proteome from flotation method-prepared granules

Dataset S7 Peptides unique to the trypsin-shaved proteome from dispersion method-prepared granules

Dataset S8 Peptides unique to the trypsin-shaved proteome from microsieve method-prepared granules

Dataset S9 Peptides unique to the trypsin-shaved proteome from flotation method-prepared granules

Dataset S10 Peptides unique to the isopropanol-solubilized proteome from dispersion method-prepared granules

Dataset S11 Peptides unique to the isopropanol-solubilized proteome from microsieve method-prepared granules

Dataset S12 Peptides unique to the isopropanol-solubilized proteome from flotation method-prepared granules

References

Heberle H, Meirelles GV, da Silva FR, Telles GP, Minghim R (2015) InteractiVenn: a web-based tool for the analysis of sets through Venn diagrams BMC Bioinformatics 16:169 doi:10.1186/s12859-015-0611-3

Mi H, Muruganujan A, Ebert D, Huang X, Thomas PD (2018) PANTHER version 14: more genomes, a new PANTHER GO-slim and improvements in enrichment analysis tools Nucleic Acids Res 47:D419-D426 doi:10.1093/nar/gky1038
